# Direct Conversion of CH_3_NH_3_PbI_3_ from Electrodeposited PbO for Highly Efficient Planar Perovskite Solar Cells

**DOI:** 10.1038/srep15889

**Published:** 2015-10-29

**Authors:** Jin-hua Huang, Ke-jian Jiang, Xue-ping Cui, Qian-qian Zhang, Meng Gao, Mei-ju Su, Lian-ming Yang, Yanlin Song

**Affiliations:** 1 Key Laboratory of Green Printing, Institute of Chemistry, Chinese Academy of Sciences Beijing 100190, P. R. China

## Abstract

Organic-inorganic hybrid perovskite materials have recently been identified as a promising light absorber for solar cells. In the efficient solar cells, the perovskite active layer has generally been fabricated by either vapor deposition or two-step sequential deposition process. Herein, electrochemically deposited PbO film is *in situ* converted into CH_3_NH_3_PbI_3_ through solid-state reaction with adjacent CH_3_NH_3_I layer, exhibiting a large-scale flat and uniform thin film with fully substrate coverage. The resultant planar heterojunction photovoltaic device yields a best power conversion efficiency of 14.59% and an average power conversion efficiency of 13.12 ± 1.08% under standard AM 1.5 conditions. This technique affords a facile and environment-friendly method for the fabrication of the perovskite based solar cells with high reproducibility, paving the way for the practical application.

Thin film solar cells are an important technology to afford cost-competitive solar energy through reduced materials and simplified fabrication process as compared to costly inorganic semiconductor-based photovoltaics^1^. Recently, organo-metal hybrid perovskites materials, such as MAPbX_3_ (MA = CH_3_NH_3_^+^; X = Cl, Br or I) have attracted tremendous attention for thin film photovoltaics due to their promising features such as solution processability, high crystallinity, direct and tunable band-gaps, and high hole/electron transport ability^3^. In the last five years, the perovskite materials have been successfully employed in both mesoscopic and planar-structured solar cells, exhibiting power conversion efficiencies (PCEs) more than 15%^4^,^5^,^6^,^7^,^8^,^9^,^10^,^11^,^12^. Recently, a certified PCE of 17.9% was reported by the National Renewable Energy Laboratory (NREL)^13^. In addition, a higher efficiency of 19.3% was also achieved through sophisticated interface engineering by Yang’s group^14^. At this stage, the identification of suitable technology for the production of perovskite solar cells with reduced wastage of toxic Pb material, low cost, and scalability to large area manufacturing would be regarded as the next important milestone.

It was found that the device performance was strongly determined by the morphology and structure of the perovskite active layer, which is in turn relied on the deposition methods^15^,^16^,^17^,^18^,^19^,^20^,^21^,^22^,^23^,^24^,^25^,^26^. In the early stages, the perovskite materials were deposited on a mesoporous or planar substrate by a single-step spin-coating method using PbX_2_ and MAX from a common solvent such as *γ*-butyrolactone^9^ or dimethylformamide^10^. In the process, rapid reaction between the two precursors happened, and resulted in the uncontrolled perovskite precipitation and large morphological variations, leading to a large fluctuation of the device performance. Considering this problem, vapor deposition technique, including dual-source or sequential method, was used to create highly flat and uniform perovskite films, and the resultant devices exhibited high PCEs of more than 15%^11^,^24^. This technique, however, required high vacuum and severe control of the operating parameters, hindering large-scale production. On the other hand, two-step approaches have been successfully investigated to prepare the perovskite film with relatively high surface coverage. The methods include two-step sequential solution process^10^, vapor assisted solution method^27^, and solution-processed precursor stacking method^28^,^29^. In the above two-step methods, PbI_2_ was first spin-coated on a mesoporous or planar substrate, followed by liquid-solid, gas-solid or solid-solid interdiffusion reaction with MAX. However, it is still a challenge to prepare a large-area, flat and uniform perovskite film using spin-coating technique, which is prerequisite for highly efficient solar cells.

In the previous report, we used an electrchemical method to deposit PbO in mesoscopic TiO_2_ films, following with iodination for PbI_2_, and interdiffusion reaction with CH_3_NH_3_I. The as-prepared CH_3_NH_3_PbI_3_ was used as a light absorber in the mesoscopic solar cells, exhibiting a high PCE of 12.5%^30^. In this work, we report that the perovskite can be successfully prepared through direct conversion of the electrodeposited PbO on *c-*TiO_2_-coated FTO glass substrate by reacting with adjacent CH_3_NH_3_I layer, allowing for a large-scale flat and uniform thin film with fully substrate coverage. The resultant planar heterojunction photovoltaic device yields a best power conversion efficiency of 14.59% and an average power conversion efficiency of 13.12 ± 1.08% under standard AM 1.5 conditions.

Electrochemical deposition is a versatile technique for producing surface coatings, possessing precise controllability, low temperature operation, rapid deposition rate, large-scale production capacity, and relatively low cost^31^,^32^,^33^,^34^,^35^,^36^. Here, we report a facile and efficient one-step method for the fabrication of the perovskite CH_3_NH_3_PbI_3_ film starting from electrodeposited PbO for efficient planar solar cells. As shown in Fig. 1, PbO is first electrochemically deposited on a fluorine-doped tin oxide glass substrate coated with a thin compact TiO_2_ (*c-*TiO_2_), followed by spin-coating a layer of MAI (CH_3_NH_3_I). The staggered layers were subjected to solid-solid interdiffusion reaction at 150 ^o^C for 1 h under N_2_ atmosphere, directly *in situ* converting to CH_3_NH_3_PbI_3_. Unlike the spin-coating method starting with PbI_2_, the film thickness of electrodeposited PbO can be well controlled, exhibiting a large-area, flat and uniform film, while certain thick PbI_2_ film was usually realized by spin coating hot and high concentration of PbI_2_ solution on the hot substrate, resulting in an unsmooth film. In addition, the PbO film could be cemented with the *c-*TiO_2_ film, favoring the charge transfer from the converted perovskite to the TiO_2_ film. Moreover, a certain amount of PbO can be deposited on the substrate as required, without the waste of toxic Pb-containing materials inevitably generated in the existing preparation techniques^6^,^10^,^11^,^23^. Using our new method, the resultant device yielded a best PCE of 14.59% under standard AM 1.5 conditions, while a PCE of 12.55% was achieved for the film prepared by the reported solution-processed precursor stacking method^28^.

Electrodeposition of metal oxide films is usually conducted by cathodic reactions in the aqueous solutions^31^,^32^,^33^,^34^,^35^. The formation mechanism of the metal oxide films was postulated as follows: base ion (OH^−^) is generated in cathode, then reacts with metal cation, such as Pb^2+^, to form corresponding metal hydroxide Pb(OH)_2_, and finally converted to PbO by dehydration^31^,^33^,^34^. Here, the electrodeposition of PbO film was performed in a single-compartment cell equipped with two electrodes: *c-*TiO_2_-coated FTO glass as a working electrode, Pt as a counter-electrode. The deposition baths consisted of aqueous solutions of 2 mM Pb(CH_3_COO)_2_, 1.5 M dimethyl sulfoxide (DMSO) and 200 mM H_2_O_2_. The electrodeposition was carried out at 70 °C with a potential of around 1.3 V. The deposited films were rinsed with water and dried in air at room temperature.

The morphology and structure of the perovskite film is crucial for the device performance. Figure 2 presents cross-sectional and top-view images of the electrodeposited PbO and *in situ* converted perovskite on the FTO-glass coated with an 80-nm-thick compact TiO_2_ (*c-*TiO_2_) layer. As shown in Fig. 2a, the as-deposited PbO film has smooth surface with full surface coverage over a large area. The high resolution image in Fig. 2b shows that a lot of holes were homogeneously distributed over the whole film with size of ~20 nm, which might form from the gas evolution during the deposition process^34^ The cross sectional image shows that the PbO film is flat over a long range with thickness of ~70 nm, as shown in Fig. 2c,d. After the reaction with CH_3_NH_3_I, the perovskite film formed showing smooth surface with crystal grain sizes ranging from 300 ~ 800 nm (Fig. 2e,f). The cross sectional images (Fig. 2g,h) show that the perovskite film is flat with thickness of ~350 nm, which is 5 times thicker than that of the corresponding PbO film due to the volume expansion as a result of interdiffusion reaction between the PbO and CH_3_NH_3_I. According to calculations, the perovskite (density: 4.16 g/cm^3^) would be about 6.3 times as thick as the lead oxide (density: 9.50 g/cm^3^) if the latter is completely converted to the former on a flat substrate with the same cross-sectional area. The actual thickness of the perovskite is lower as compared to the calculated value, which is most likely due to the presence of the holes in the PbO film (Fig. 2b).

X-ray diffraction (XRD) was used for the phase identification of the electrodeposited PbO and *in situ* converted perovskite films on the *c-*TiO_2_/FTO substrate. XRD pattern of the *c-*TiO_2_/FTO substrate was recorded for comparison, as shown in Fig. 3a. After the electrodeposition, new peaks appeared at 28.2°, 32.7°, 46.9°, and 55.7°, which are assigned to [111], [200], [220], and [311] of lead oxide crystal planes, corresponding to the data in JCPDS card no. 27–1201. After coating CH_3_NH_3_I on the PbO surface and heating at 150 ^o^C for 30 minutes, the peaks for the PbO disappeared, and new peaks were observed. The peaks located at 12.6°, 34.3°, 39.5°, and 52.3° are assigned to (001), (012), (003), and (004) lattice planes of the 2H polytype PbI_2_ (JPCDS card No. 73–1750)^10^, while the peaks at 14.2°, 20.0°, 23.6°, 24.6°, 28.5°, 31.9°, 35.0°, 40.6°, and 43.2°, are corresponding to the reflections from [110], [200], [211], [202], [220], [310], [312], [224], and [314] of the perovskite^27^. After the reaction lasted for 1 h, the peaks for PbI_2_ disappeared, and the intensity of the peaks from the perovskite became stronger without other new peaks, indicating the complete transformation from the electrodeposited PbO to the perovskite CH_3_NH_3_PbI_3_ through the *in situ* solid-solid reaction. The phase evolution indicates the formation mechanism of the perovskite as follows:

The CH_3_NH_3_I gradually decomposed to CH_3_NH_2_ and HI at the elevated temperature at the initial stage (Fig. 4a), and the generated HI further reacted with the PbO to form PbI_2_ (Fig. 4b). Finally the PbI_2_ reacted with CH_3_NH_3_I to form the perovskite CH_3_NH_3_PbI_3_ (Fig. 4c), similar to the Huang’s report^27^. In the processes, PbI_2_ is an intermediate. It should be noted that equivalent amount of H_2_O was generated during the conversion process from PbO to PbI_2_ (Eq-b), and the water could have positive effect on the formation of the provskite layer and thus improve the photovoltaic performance^37^,^38^.

XPS was used to determine the chemical environment and stoichiometry at the surface of the perovskite film. Pb 4f and I 3d core level spectra were shown in Fig. 5, and the whole spectra were presented in Fig. S1. In the case of Pb 4f, the binding energies of Pb 4f_7/2_ and Pb 4f_5/2_ were observed at 138.40 eV and 143.37 eV with a spin−orbit split of 4.97 eV. For I 3d, the energies of I 3d_5/2_ and I 3d_3/2_ were found at 619.49 eV and 630.88 eV with a spin−orbit split of 11.39 eV. All the data are well consistent with those reported previously for CH_3_NH_3_PbI_3_ film^36^. In addition, the stoichiometry between lead and iodine was roughly assessed to be about 1:3 through the integrated intensity of the peaks for Pb 4f and I 3d core levels. For further investigation of the evolution, EDX mapscan was performed for the cross sectional perovskite film, as shown in Fig. S2. The result shows that the atomic ratio for lead and iodine is about 1:3 for the CH_3_NH_3_PbI_3_ film. In combination with the XRD results, it is believed that the electrodeposited PbO film could be completely *in situ* converted to the perovskite through solid-solid interdifussion reation with CH_3_NH_3_I. Recently, PbO coated TiO_2_ films were fabricated by calcination of the film prepared from a mixture of TiO_2_ colloid and PbI_2_ or Pb(CH_3_COO)_2_. The as-prepared PbO on the films were further transferred to PbI_2_ through reaction with HI. The resultant PbI_2_-coated TiO_2_ films were used for the preparation of the perovskite-decorated TiO_2_ films. In the reports, it was observed that the perovskite had poor coverage on TiO_2_ particles or nanofibers in the films^39^,^40^.

In our experiments, the resultant perovskite film is flat and uniform with full surface coverage, which is prerequisite for high efficient perovskite solar cells. We also tried to prepare PbI_2_ film by spin coating method, and then *in situ* being converted to the perovskite according to the reported method^27^. We found it difficult to get a flat and uniform perovskite film (as shown in Fig. S3). In addition, other soluble Pb-salts such as Pb(CH_3_COO)_2_, Pb(NO_3_)_2_, Pb(ClO_4_)_2_ can be used for the electrodeposition of PbO film^31^,^32^,^33^,^34^. In the electrodeposition, they can be completely converted to the PbO without waste. Thus, the present method is environment-friendly for the fabrication of the perovskite active film for the photovoltaic devices.

The resultant perovskite film was used as light harvester for fabrication of planar heterojunction solar cells. The device (denoted as **device 1**) was constructed with a structure of FTO/*c-*TiO_2_ (~80 nm)/CH_3_NH_3_PbI_3_ (~350 nm)/Spiro-OMeTAD (150 nm)/Au (80 nm) (see Fig. S4). For comparison, another device was prepared with the same architecture except for the perovskite active layer fabricated starting from PbI_2_ according to the previous report (denoted as **device 2**)^28^. Both the devices were characterized by recording photocurrent density–voltage (*J−V*) curves with reverse (from *V*_*oc*_ to *J*_*sc*_) or forward (from *J*_*sc*_ to *V*_*oc*_) bias scanning at a rate of 100 mV/s under AM 1.5 irradiation (100 mW cm^−2^), and the photovoltaic parameters were listed in Table 1. As shown in Fig. 6a, the best performing **device 1** gave a short circuit photocurrent density (*J*_*sc*_) of 20.97 mA/cm^2^, an open circuit voltage (*V*_*oc*_) of 0.98 V, a fill factor (*FF*) of 0.71, and a power conversion efficiency (PCE) of 14.59% with the reversed scaning, and the average PCE of **device 1** is 13.12% for 16 samples with a relatively low standard deviation of 8% (shown in Fig. S6a and Table S1). In the case of the reverse bias scanning, slight hysteresis effect was observed for the **device 1**, where the best perorming **device1** gave a relatively lower PCE of 13.90% due to the decreased FF (0.69 vs. 0.71). With the reverse bias scanning, the best performing **device 2** gave a *J*_*sc*_ of 19.81 mA/cm^2^, a *V*_*oc*_ of 0.96 V, a *FF* of 0.66, and a PCE of 12.55%, with an average PCE of 9.96% and a standard deviation of 15%, as shown in Fig. S5, S6b and Table S2. In addition, obvious hysteresis effect was observed for the **device 2**, where the best performing **device 2** gave a decreased PCE of 11.2% under the forward scanning. The results indicate the **device 1** has a higher PCE and reproducibility as compared to the **device 2**, which can be explained due to the high-quality perovskite film evolved from the flat and uniform eletrodeposited PbO film. Figure 6b shows the external quantum efficiency (*EQE*) spectrum of **device 1**. A broad spectral response in the range of 300–820 nm was observed with the highest value of 82% at 530 nm. The integrated current density (20.44 mA/cm^−2^) is well consistent with the measured value of the *J*_*sc*_.

In summary, we developed a facile and environment-friendly technology for the fabrication of long-range continuous and homogeneous perovskite CH_3_NH_3_PbI_3_ film with full surface coverage. In the method, electrodeposited PbO films can be *in situ* directly converted to the perovskite through solid-solid interdiffusion reation with CH_3_NH_3_I. We proposed a plausible mechanism for the formation of uniform and dense provskite layer. The resulting perovskite film was used as light harvester to construct planar heterojunction solar cells, giving an initial power conversion efficiency of 14.59%, which is among the highest values. Further efficiency enhancement can be expected following the optimization of the film thickness, morphology and relative interface engineering. The combination of simple control of deposit thickness, low processing temperature, low cost of equipment, large-scale, high reproducibility and environmental friendly process, will make this method as a promising technique for the practical production of the perovskite and other thin film photovoltaics.

## Methods

Substrate preparation was carried out under ambient conditions. FTO-coated glass (14 ohm/sq) was patterned by etching with Zn powder and 2 M HCl diluted in deionized water. The substrates were then cleaned with detergent diluted in deionized water, rinsed with deionized water, acetone and ethanol, and dried with clean dry air. After oxygen plasma treatment, the clean substrates was spin-coated with 0.15 M and 0.3 M titanium diisopropoxide bis(acetylacetonate) at 3,000 r.p.m. for 30 s subsequently. After drying at 125 °C for 10 min, they were sintered at 500 °C for 60 min in air. The substrate was immersed in 40 mM TiCl_4_ aqueous solutions at 70 °C for 30 min and washed with distilled water and ethanol, followed by annealing at 500 °C for 30 min in air to form a compact layer of TiO_2_ (*c-*TiO_2_).

The lead oxide film was prepared according to the following method: Electrodeposition of thin films was performed in a single-compartment cell equipped with two electrodes: FTO/*c-*TiO_2_ glass as a working electrode, Pt as a counter-electrode. The deposition baths consisted of aqueous solutions of 2 mM Pb(CH_3_COO)_2_, 1.5 M DMSO and 200 mM H_2_O_2_ (It needs to bubble sufficient nitrogen gas to displace the dissolved air before adding H_2_O_2_). The lead oxide film was carried out by potentiostatic electrolysis, the thickness and quality of the film is optimized by controlling the deposition parameters such as time, deposition potential (1.3 V), the deposition temperature (70 °C), etc. The deposited films were rinsed with water and dried under air at room temperature. After drying, 30 μL MAI solutions (50 mg/mL) were spun on the top of the film at 4,000 rpm for 30 s. The films were placed in a petridish and annealled at 150 °C for 1 h. After cooling down, the isopropanol was spun on the as-prepared films at 4,000 rpm for 30 s and dried at 70 °C. then, the spiro-OMeTAD-based hole-transfer layer (80 mg spiro-OMeTAD, 28.5 μL 4-tertbutylpyridine and 17.5 μL lithium-bis(trifluoromethanesulfonyl) -imide (Li-TFSI) solution (520 mg Li-TFSI in 1 ml acetonitrile) all dissolved in 1 ml chlorobenzene) was deposited by spin coating at 4,000 r.p.m. for 30 s. Finally, the counter Au electrode was deposited by thermal evaporation at a base pressure of 5 × 10^−5^ mbar. The active area was 0.04 cm^2^.

## Additional Information

**How to cite this article**: Huang, J.-H. *et al.* Direct Conversion of CH_3_NH_3_PbI_3_ from Electrodeposited PbO for Highly Efficient Planar Perovskite Solar Cells. *Sci. Rep.* **5**, 15889; doi: 10.1038/srep15889 (2015).

## Supplementary Material

Supplementary Information

## Figures and Tables

**Figure 1:**
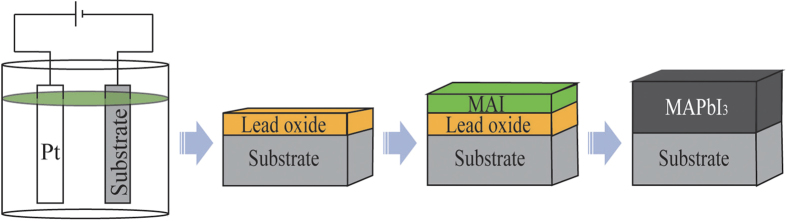
Schematic illustration of the perovskite CH_3_NH_3_PbI_3_ film formation on *c*-TiO_2_ coated FTO glass substrate starting from electrodeposited PbO layer and subsequent *in-situ* reaction with adjacent CH_3_NH_3_I (MAI) layer.

**Figure 2:**
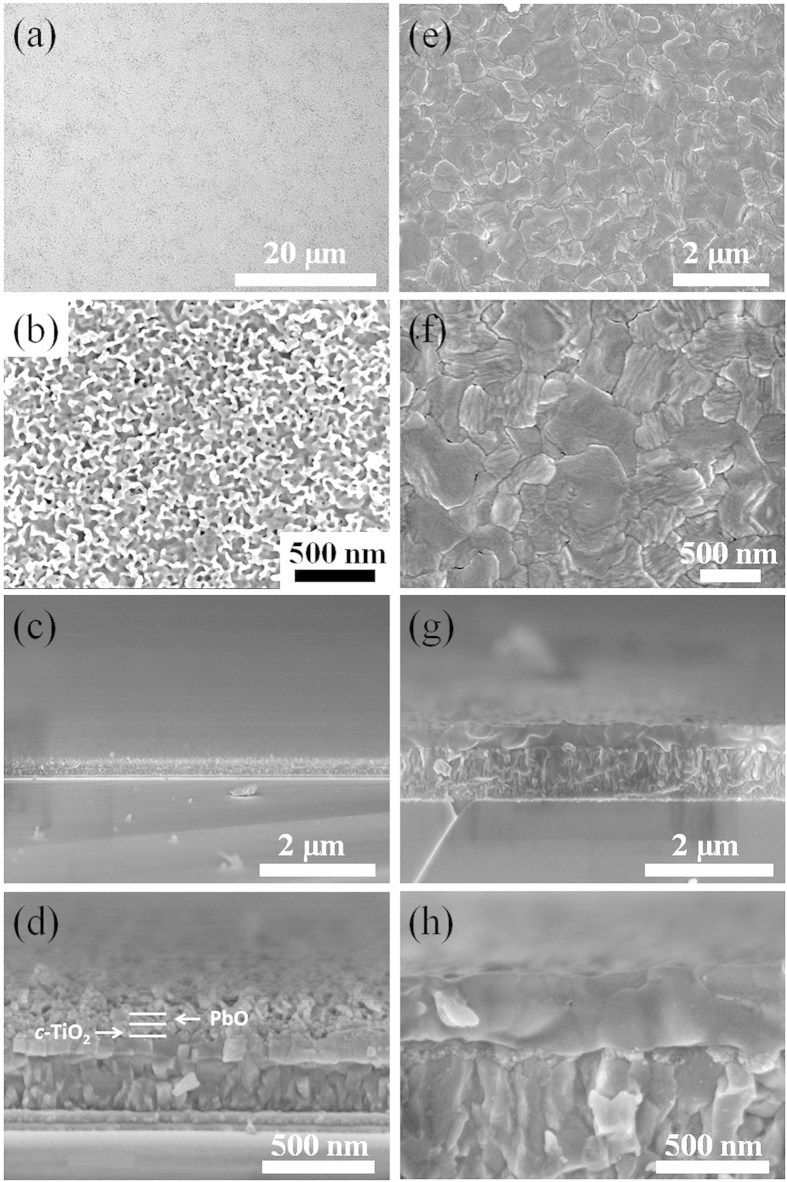
Top-view SEM images of electrodeposited lead oxide film, (a) with low magnification, (b) with high magnification. Cross-sectional SEM images of the lead oxide film on *c*-TiO_2_ coated FTO, (c) with low magnification, (d) with high magnification. Top-view SEM images of the perovskite film *in situ* prepared from the reaction of the lead oxide and MAI, (**e**) with low magnification, (**f**) with high magnification. Cross-sectional SEM images of the perovskite film on *c*-TiO_2_ coated FTO, (**g**) with low magnification, (**h**) with high magnification.

**Figure 3:**
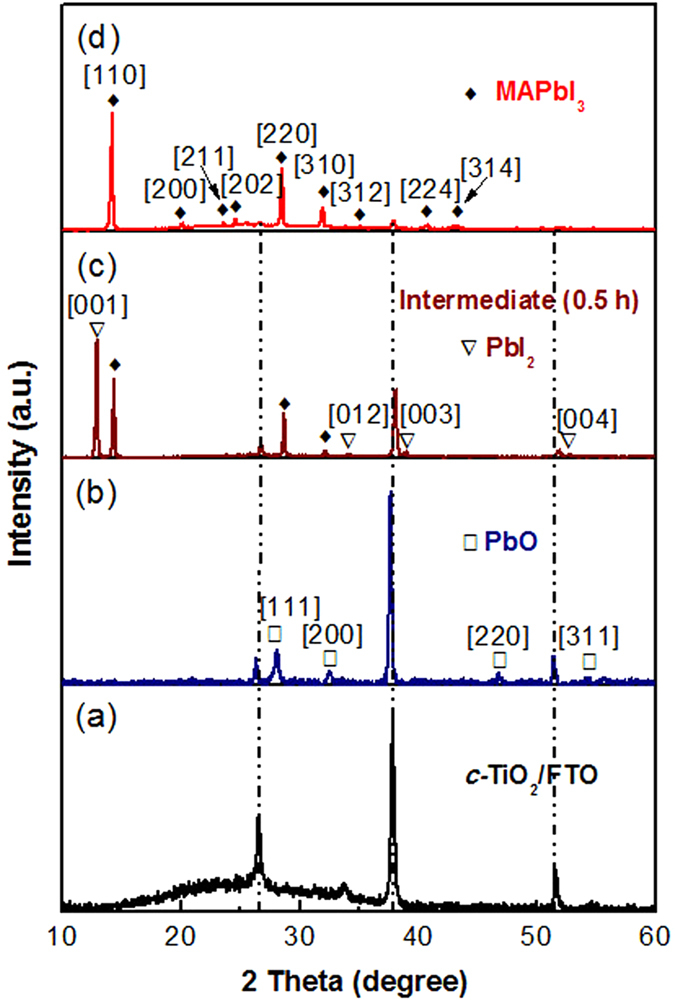
The XRD patterns of (a) the *c-*TiO_2_/FTO substrate, (b) the electrodeposited PbO on the substrate, (c) the intermediate on the substrate after 0.5 h interdiffusion reaction, and (d) the perovskite film on the substrate after 1 h interdiffusion reaction.

**Figure 4:**
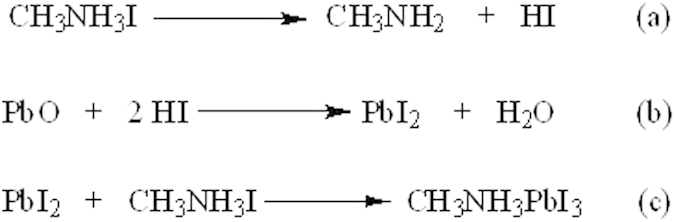
The mechanism to form the perovskite from PbO and CH3NH3I.

**Figure 5:**
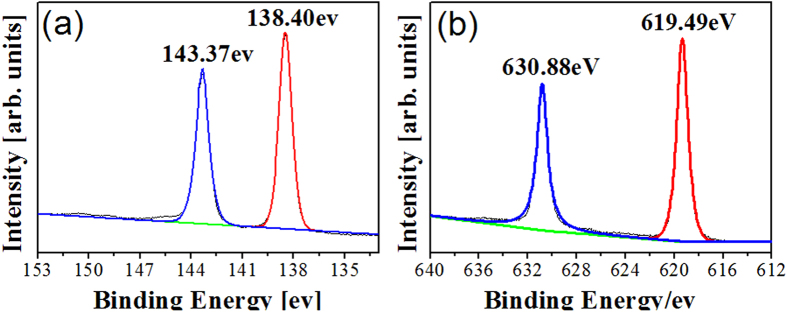
Pb 4f (**a**) and I 3d (**b**) core level spectra of the as-prepared perovskite surface measured with a photonenergy of 4000 eV.

**Figure 6:**
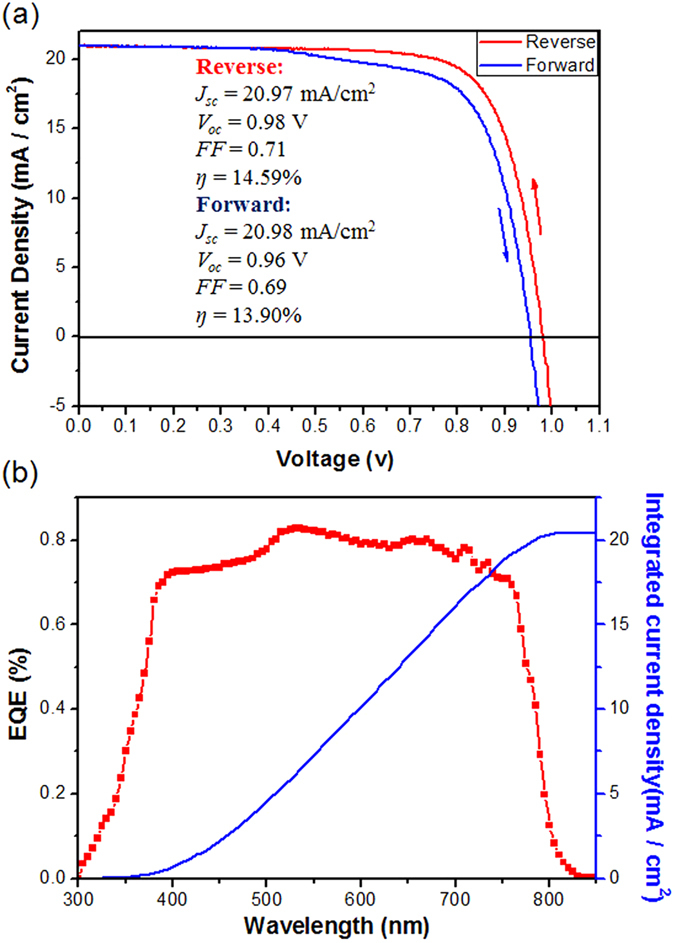
(**a**) *J-V* characteristics of device 1 with reverse (from **V**_oc_ to **J**_sc_) or forward (from **J**_sc_ to **V**_oc_) bias scanning at a rate of 100 mV/s under AM 1.5 irradiation (100 mW cm^−2^) under AM 1.5 G illumination; (**b**) the *EQE* spectrum (red) and the integrated photocurrent density (blue) of device 1 expected to be generated under AM 1.5 G irradiation.

**Table 1:** Photovoltaic performance of **device 1** and **device 2** measured with reverse and forward bias scanning at a rate of 100 mV/s under AM 1.5 irradiation (100 mW cm^−2^) under the 100 m W cm^−2^ AM1.5G illumination.

Device		J_sc_/mA cm^−2^	V_oc_/V	FF	PCE/%
*1*	Rev.^[a]^	20.97	0.98	0.71	14.59
	For.^[b]^	20.98	0.96	0.69	13.90
	av.^[c]^	19.81 ± 1.57	0.96 ± 0.03	0.69 ± 0.02	13.12 ± 1.08
*2*	Rev.^[a]^	19.81	0.96	0.66	12.55
	For.^[b]^	19.85	0.91	0.62	11.20
	av.^[c]^	17.82 ± 2.18	0.90 ± 0.05	0.62 ± 0.13	9.96 ± 1.52

^[a]^reverse scan; ^[b]^forward scan; ^[c]^average and standard deviation for a batch of 16 samples.
