# Supplementary material for: Direct Conversion of CH3NH3PbI3 from Electrodeposited PbO for Highly Efficient Planar Perovskite Solar Cells
Source: Sci Rep. 2015 Oct 29;5:15889. doi: 10.1038/srep15889 (PMC4625132; doi:10.1038/srep15889)
Supplement: Supplementary Information [file srep15889-s1.doc]

Direct Conversion of CH_3_NH_3_PbI_3_ from Electrodeposited PbO for Highly Efficient Planar Perovskite Solar Cells

Jin-hua Huang, Ke-jian Jiang,* Xue-ping Cui, Qian-qian Zhang, Meng Gao, Mei-ju Su, Lian-ming Yang, Yanlin Song*

Key Laboratory of Green Printing, Institute of Chemistry, Chinese Academy of Sciences, Beijing 100190, P. R. China.


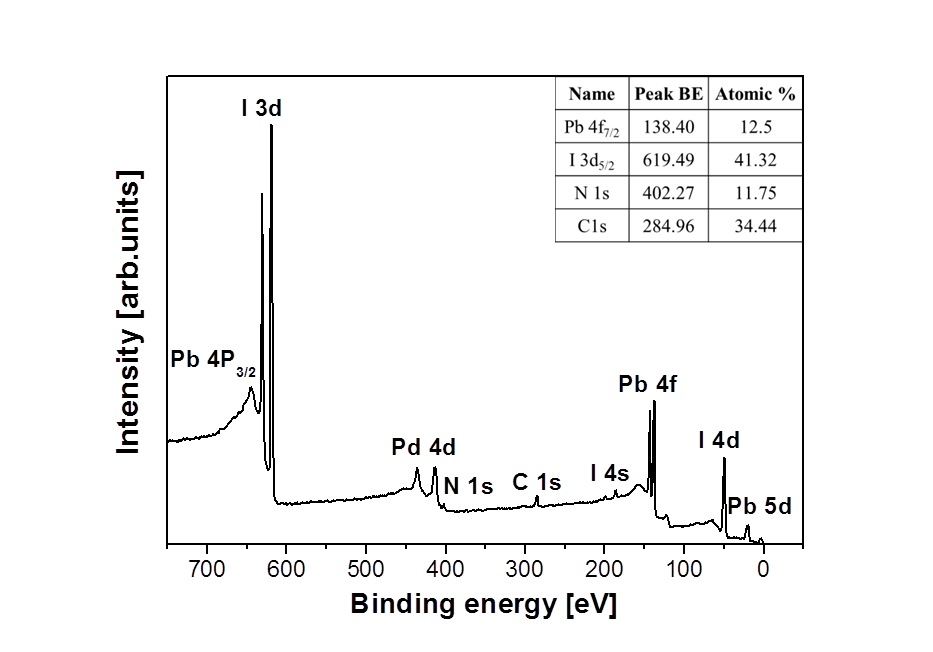


**Figure [S1].** The XPS surface analysis of the as-prepared perovskite surface measured with a photonenergy of 4000 eV, the inset showed the atomic percentages of the corresponding elements.


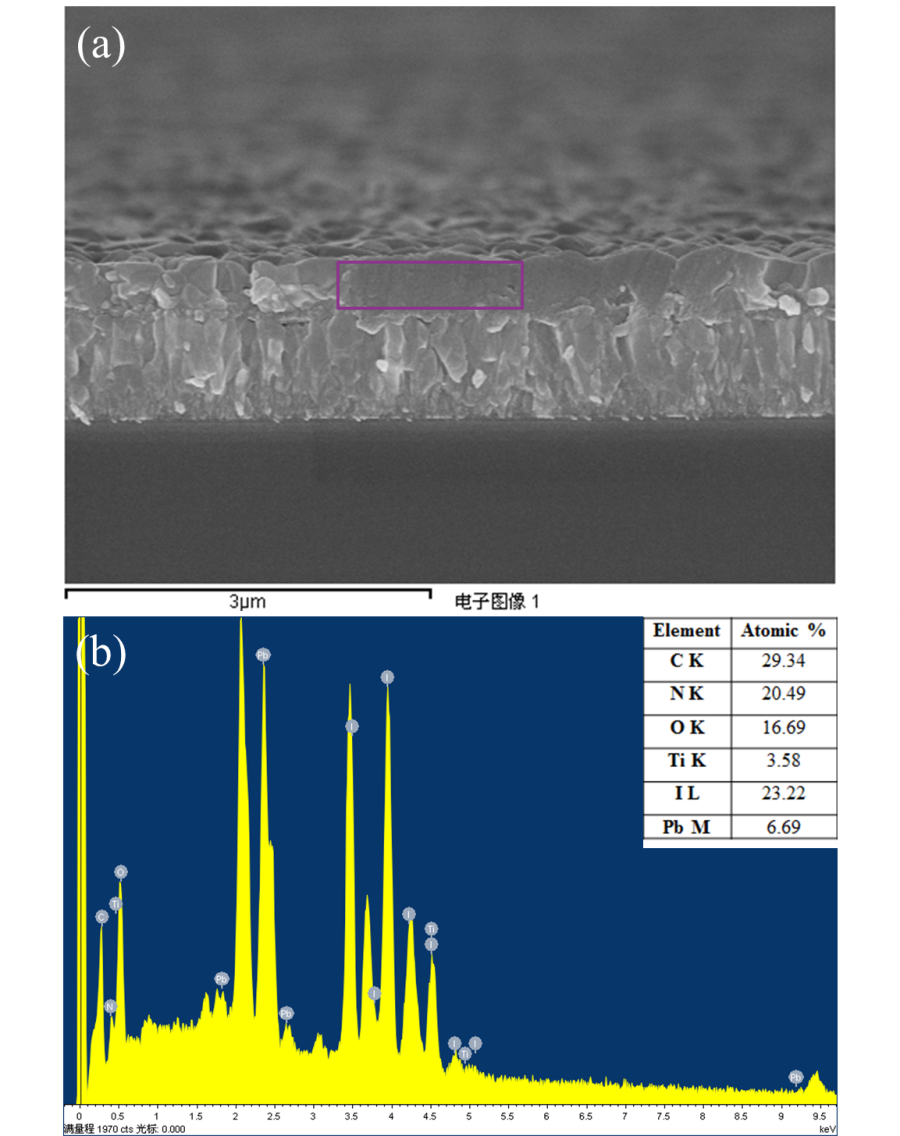


**Figure [S2].** The SEM-EDX analysis of the as-prepared CH_3_NH_3_PbI_3_ film: (a) Cross-sectional SEM image of the film, (b) EDX patterns for the area shown in (a); the inset showed the atomic percentages of the corresponding elements.


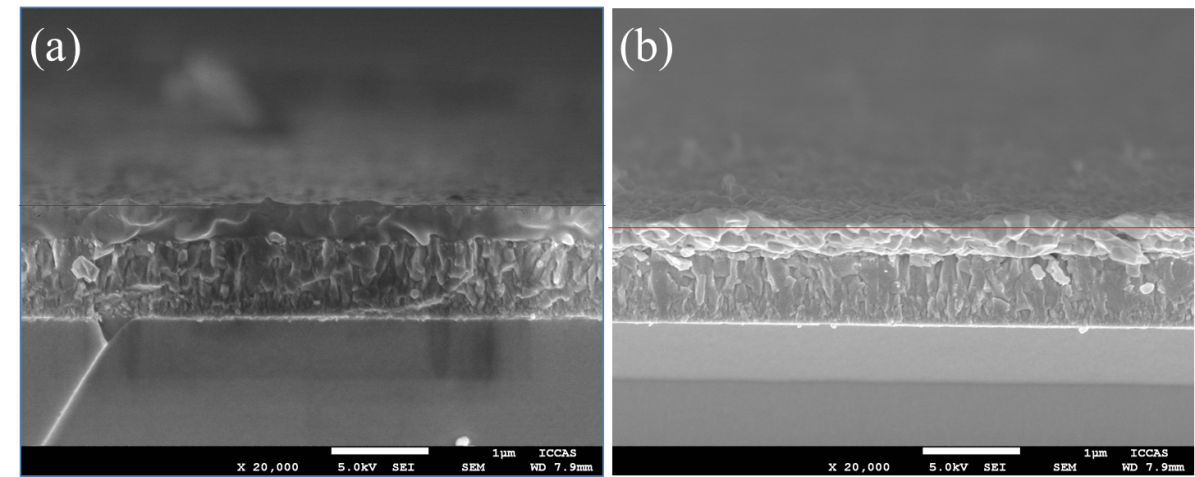


**Figure [S3].** The Cross-sectional SEM images of the perovskite film prepared from the PbO **(**a**)** and the PbI_2_ film (b).


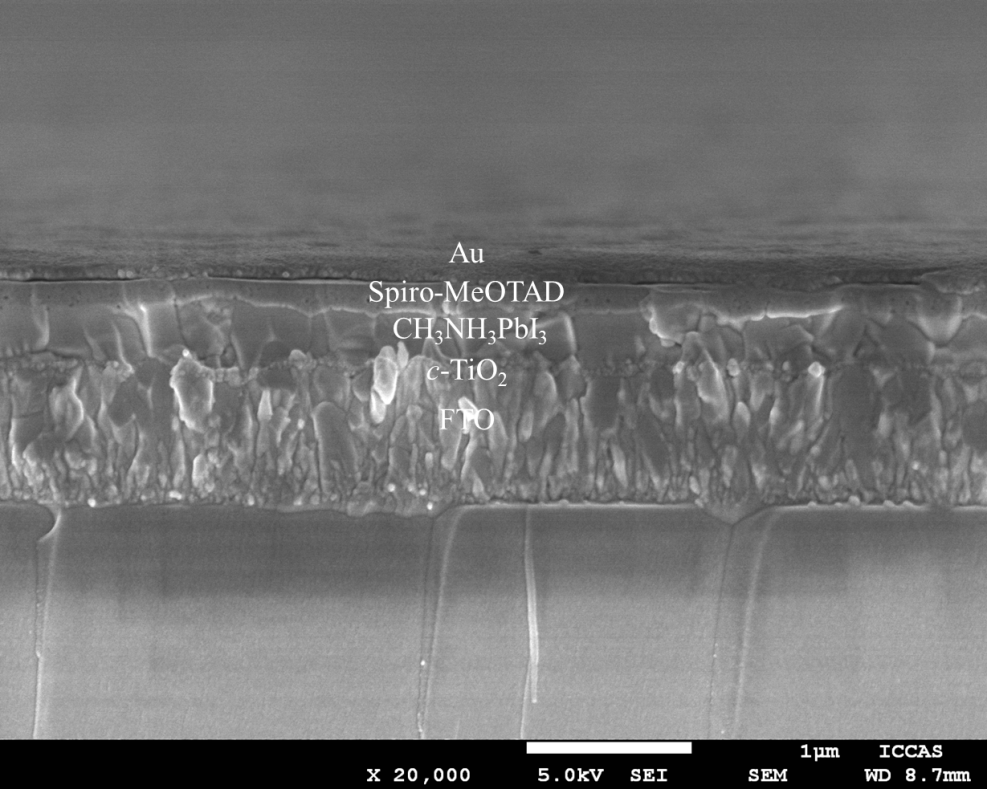


**Figure [S4].** The cross-sectional SEM image of the **device 1**: FTO/*c-*TiO_2_ (~ 80 nm)/ CH_3_NH_3_PbI_3_ (~350 nm) /Spiro-OMeTAD (150 nm) /Au (80 nm).


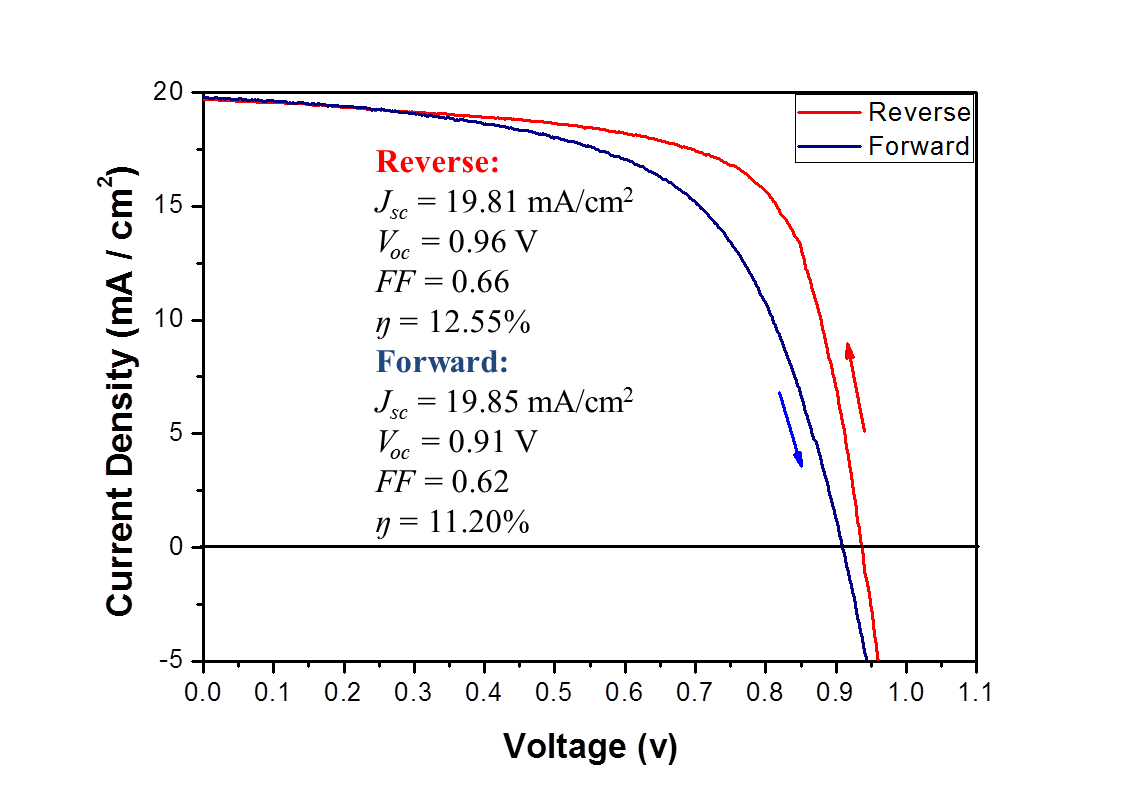


**Figure** **[S5].** *J−V* characteristics of **device 2** with reverse (from *V_oc_* to *J_sc_*) or forward (from *J_sc_* to *V_oc_*) bias scanning at a rate of 100 mV/s under AM 1.5 irradiation (100 mW cm^-2^) under AM 1.5G illumination.


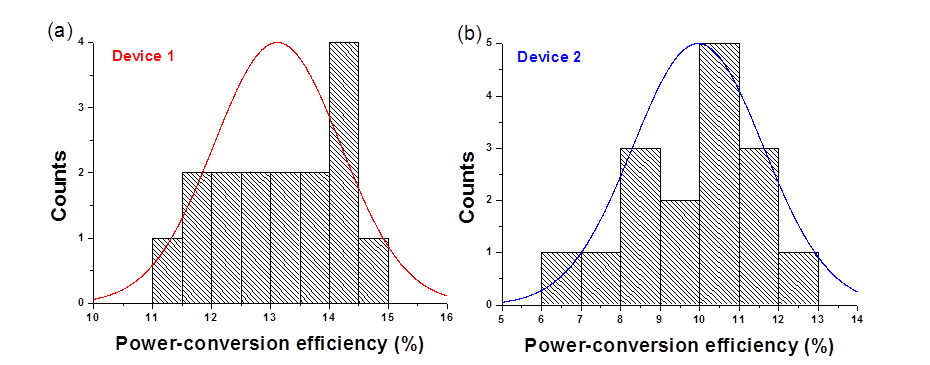


**Figure [S6].** The statistic distribution of the **device 1** and **device 2**.

**Table S1.** Photovoltaic parameters of **device 1** for a batch of sixteen samples measured under 100 mW cm^-2^ simulated AM1.5G illumination.

| cell | *J_sc_*  (mA/cm^2^) | *V_oc_*  (V) | *FF* | *PCE*  (%) |
| --- | --- | --- | --- | --- |
| 1 | 17.81 | 0.97 | 0.68 | 11.75 |
| 2 | 20.55 | 0.98 | 0.70 | 14.10 |
| 3 | 18.94 | 0.96 | 0.67 | 12.18 |
| 4 | 18.63 | 0.99 | 0.63 | 11.62 |
| 5 | 21.67 | 0.95 | 0.69 | 14.21 |
| 6 | 21.39 | 0.93 | 0.72 | 14.32 |
| 7 | 20.47 | 0.99 | 0.68 | 13.78 |
| 8 | 17.98 | 1.01 | 0.71 | 12.89 |
| 9 | 17.49 | 0.93 | 0.69 | 11.22 |
| 10 | 20.97 | 0.98 | 0.71 | 14.59 |
| 11 | 21.89 | 0.91 | 0.66 | 13.15 |
| 12 | 18.11 | 0.96 | 0.69 | 12.00 |
| 13 | 21.21 | 0.96 | 0.69 | 14.05 |
| 14 | 20.56 | 0.95 | 0.70 | 13.67 |
| 15 | 18.20 | 0.97 | 0.73 | 12.89 |
| 16 | 21.01 | 0.94 | 0.68 | 13.43 |
| Average | 19.81± 1.57 | 0.96± 0.03 | 0.69±0.02 | 13.12± 1.08 |

**Table S2.** Photovoltaic parameters of **device 2** for a batch of sixteen devices measured under 100 mW cm^-2^ simulated AM1.5G illumination.

| Cell | *J_sc_*  (mA/cm^2^) | *V_oc_*  (V) | *FF* | *PCE*  (%) |
| --- | --- | --- | --- | --- |
| 1 | 19.51 | 0.97 | 0.63 | 11.92 |
| 2 | 15.67 | 0.95 | 0.55 | 8.19 |
| 3 | 18.46 | 0.91 | 0.61 | 10.25 |
| 4 | 14.54 | 0.86 | 0.61 | 7.63 |
| 5 | 17.32 | 0.95 | 0.57 | 9.38 |
| 6 | 19.21 | 0.83 | 0.68 | 10.84 |
| 7 | 19.81 | 0.96 | 0.66 | 12.55 |
| 8 | 14.7 | 0.95 | 0.68 | 9.50 |
| 9 | 13.58 | 0.91 | 0.56 | 6.92 |
| 10 | 19.11 | 0.93 | 0.58 | 10.31 |
| 11 | 19.89 | 0.93 | 0.59 | 10.91 |
| 12 | 17.32 | 0.81 | 0.60 | 8.42 |
| 13 | 18.88 | 0.87 | 0.65 | 10.68 |
| 14 | 16.84 | 0.83 | 0.62 | 8.67 |
| 15 | 20.13 | 0.89 | 0.63 | 11.29 |
| 16 | 20.15 | 0.88 | 0.67 | 11.88 |
| Average | 17.82± 2.18 | 0.90± 0.05 | 0.62± 0.13 | 9.96± 1.52 |
